# Clinical variability of equine asthma phenotypes and analysis of diagnostic steps in phenotype differentiation

**DOI:** 10.1186/s13028-024-00773-7

**Published:** 2024-09-18

**Authors:** Lia Kristin Meiseberg, Julien Delarocque, Nicole de Buhr, Bernhard Ohnesorge

**Affiliations:** 1 Clinic for Horses, University of Veterinary Medicine Hannover, Foundation, Bünteweg 9, 30559 Hannover, Germany; 2 Institute of Biochemistry, University of Veterinary Medicine Hannover, Foundation, Bünteweg 17, 30559 Hannover, Germany; 3 Research Center for Emerging Infections and Zoonoses (RIZ), University of Veterinary Medicine Hannover, Foundation, Bünteweg 17, 30559 Hannover, Germany

**Keywords:** Bronchoalveolar lavage, Chronic obstructive bronchiolitis, Equine asthma, Phenotype

## Abstract

**Background:**

Equine asthma is a common, non-infectious, chronic lung disease that affects up to 80% of the horse population. Strict phenotyping and identification of subclinically asthmatic horses can be challenging. The aim of this study was to describe equine asthma phenotypes (mild, moderate, and severe asthma) defined by BALF cytology and occurrence of clinical signs in a population of privately owned horses and to identify the variables and examination steps with best discriminative potential. The standardised examination protocol included clinical examinations, blood work, airway endoscopy with bronchoalveolar lavage fluid analysis, arterial blood gas analysis and radiography under clinical conditions performed by one veterinarian.

**Results:**

Out of 26 horses, four were diagnosed with mild (subclinical), seven with moderate, and seven with severe asthma based on clinical examination and BALF cytology. Eight horses served as controls. Cough with history of coughing was the strongest variable in phenotype differentiation. Factor analysis revealed an increasing clinical variability with disease severity and an overlapping of clinical presentations between phenotypes. Elevated mast cell (4/4 horses) and neutrophil counts (3/4 horses) in bronchoalveolar lavage cytology differentiated mild asthmatic horses from healthy horses. Moderate and severe asthmatic horses were characterised by clinical signs and neutrophil counts.

**Conclusions:**

The results indicate that medical history, clinical examination and bronchoalveolar lavage cytology are minimum indispensable steps to diagnose equine asthma and that phenotypes are clinically overlapping. A differentiation of three phenotypes without neutrophil and mast cell counts in bronchoalveolar lavage cytology is not sufficient for clinical diagnostics. A comparably exact diagnosis cannot be achieved by relying on alternative examinations used in this study. Screenings of inconspicuous horses with bronchoalveolar lavage can aid in diagnosing subclinically affected animals, however, group size was small, the procedure is invasive and clinical relevance of slightly elevated cells in bronchoalveolar lavage remains unclear. Clinical relevance could not be clarified in this study, since follow-up examinations or lung function testing were not performed.

**Supplementary Information:**

The online version contains supplementary material available at 10.1186/s13028-024-00773-7.

## Background

Equine asthma (EA) is a highly prevalent, non-infectious, chronic lung disease which is separated into the phenotypes mild-moderate EA (mEA) and severe EA (sEA). These EA phenotypes were formerly known as separate diseases, namely, inflammatory airway disease (IAD) or chronic obstructive bronchitis (COB), recurrent airway obstruction (RAO) and summer-pasture associated recurrent airway obstruction (SPA-RAO). Since 2016, EA is rather described as a spectrum of partly overlapping clinical pictures or ‘EA syndrome’, rather than discrete entities [1]. This definition is still under discussion [2]. It has been demonstrated that 60–80% of horses examined through bronchoalveolar lavage fluid (BALF) cytology are affected by mEA [3–5]. Furthermore, 14–17% of the horse population show signs consistent with sEA [5, 6]. These numbers highlight the immense impact of the disease on equine population health, with economical and psychological repercussions for the owners, but also ethical implications regarding animal welfare and husbandry practices. The Consensus Statement of the American College of Veterinary Internal Medicine (ACVIM) provided diagnostic guidelines based on cut-off values for BALF cytology and discussed the associated clinical presentations [1]. Still, phenotyping remains difficult, and a sufficient clinical differentiation of mild EA is not reliably possible. Asthmatic horses show a variety of clinical characteristics such as cough, exercise intolerance and elevated breathing effort at rest [7, 8]. Symptoms are dependent on disease severity but also on management and season of the year [4, 9]. The mild-moderate EA phenotype comprises asymptomatic and symptomatic horses. Per consensus statement definition, horses with mEA do not exhibit elevated breathing effort at rest, making it difficult to diagnose in a variety of cases. Airway inflammation is supposed to be mastocytic, mixed mastocytic-neutrophilic or neutrophilic with a maximum of 25% neutrophils in BALF [1]. At present, mEA cannot be strictly distinguished into mild or moderate EA, leading to a variety of clinical presentations under this definition. Some authors suggest to define mildly affected horses through a lack of clinical signs apart from a perceived exercise intolerance. Diagnosis is thus made in accordance with pathological BALF cytology after exclusion of other causes leading to exercise intolerance. It is mostly investigated in thoroughbred racehorses [10–13]. Since BALF analysis is not performed regularly on asymptomatic horses, mild EA might be underdiagnosed as e.g., a cause of poor performance in the horse population [8, 10, 14]. Severe EA is accompanied by airway neutrophilia (≥ 25%), marked clinical signs, insensitivity to treatment and irreversible changes of the lung tissue with disease progression [15–17]. It is not fully understood in which cases mEA progresses to sEA. Still, it could be beneficial to recognize subclinically affected horses and to improve their management early on, to eliminate possible negative influences from management and housing conditions on respiratory health. Just recently, a review on evidence for diagnosing mEA highlighted the lack of clear diagnostic guidelines for the mEA phenotype [7]. The authors underlined the high risk of bias and the need for a standardisation of diagnostic methods. They also mentioned blood biomarkers or diagnostic procedures currently under investigation, in line with the future directions of equine asthma research pointed out by the ACVIM consensus statement expert panel [1, 8].

The aim of this study was to investigate clinical findings in horses with and without signs of lower respiratory disease defined into three equine asthma phenotypes; mild EA (miEA), moderate EA (modEA) and severe EA (sEA) and to identify the most informative parameters to reach the diagnosis.

## Methods

### Animals

A diagnostic standardised protocol was offered as health and performance check-up for privately owned horses presented to the Clinic for Horses of the University of Veterinary Medicine Hannover, Foundation, between December 2021 and March 2023 to obtain comparable data for analysis. An online campaign was launched several weeks prior to the beginning of the examination period to recruit patients. Any horse free of medication for at least 14 days before presentation was considered, independently of the presence or absence of clinical signs of respiratory disorders. Horses with evidence of infectious diseases or non-respiratory disorders were excluded from the study based on clinical history, clinical examination, and complete blood count. In total, 26 horses of different breeds were included in the study (13 Hannoverians, 3 Icelandic Horses, 3 Westfalians, 1 Finn Horse, 1 German Sporthorse, 1 Holsteiner, 1 Irish Sporthorse, 1 Oldenburger, 1 P.R.E., and 1 Quarter Horse). All examinations were conducted to confirm or exclude respiratory disease and were performed at the owners’ demand. Owners gave written informed consent for the further usage of data and samples.

### Study design

All horses were examined for general and respiratory health. Medical and housing history was obtained from the owners using a questionnaire (Additional File 1). All horses arrived one day prior to examinations to acclimate to surroundings. They were kept in a box stall on wood shavings with 24-h access to a concrete-floored paddock and were given washed hay while in hospital. The day after presentation, all horses were examined, and the findings were used to score the horses for general health by one veterinarian (Table 1).

**Table 1:** Anamnestic and clinical scoring system used to evaluate asthma severity in the study horse population

Clinical sign or symptom reported	Score	Comment
Nasal discharge	0	None or light serous discharge
	1	Yes (e.g., mucous, purulent)
Cough and history of coughing	0	None
	1	Not at examination but single coughs noticed by owners the past weeks
	2	Not at examination but single coughs regularly daily or always at start of exercise noticed by owners the past weeks
	3	Cough spontaneously at examination or cough attacks noticed by the owner the past weeks
Dyspnoea at rest	0	No dyspnoea (costo-abdominal breathing pattern)
	1	Slightly elevated abdominal lift at end of expiration
	2	Moderate abdominal lift without other signs of elevated breathing effort
	3	Severe abdominal breathing pattern, inspiratory flared nostrils
	4	Severe dyspnoea with pendular body movement, nostril flaring, signs of general discomfort
Auscultation	0	Physiological (soft inspiratory respiratory noise)
	1	Mildly forced inspiratory respiratory noise
	2	Moderately forced inspiratory and mild expiratory vesicular noises
	3	Severely forced in- und expiratory respiratory noises and/or tracheal rattling noises
	4	Rhonchus or rattling noises
*Individual score (max. 12)*		

Medical history was included as part of the cough score, since cough was considered an objective symptom to be noticed reliably by the owners, if horses are regularly supervised. All horses were observed on a daily basis by their owners or caretakers in this study. The scoring system aimed at objectifying clinical examination results and history of cough for further analysis. The anamnestic and clinical score is in the following referred to as ‘clinical score’

A blood sample was obtained for differential blood cell counts (ADVIA® 120 Hematology System, Siemens Healthcare GmbH, Erlangen, Germany), and measurement of electrolytes, total plasma protein and fibrinogen concentrations. Arterial blood (2 mL) was drawn via puncture of the A. transversa faciei or the A. carotis communis into electrolyte-balanced heparinized plastic syringes (PICO50®, Radiometer GmbH, Krefeld, Germany) and directly analysed with a blood-gas analyser (ABL825 Flex®, Radiometer GmbH, Krefeld, Germany). Radiographs of the lungs (cranioventral and caudodorsal view) were taken after sedation of the horses with detomidine and butorphanol (Cepesedan® 0.01 mg/kg BW and Butorgesic® 0.01 mg/kg BW i.v., CP-Pharma, Burgdorf, Germany). The radiographs were screened for abnormalities by a boarded veterinarian to exclude any case indicative of thoracal masses or equine multinodular pulmonary fibrosis (EMPF). Endoscopic examination (Olympus, SIF Q140, Olympus Europa SE & Co. KG, Hamburg, Germany) was started directly after radiography. The horses were restrained in stocks and top-up boluses of detomidine administered intravenously (0.005 mg/kg BW) as necessary. Findings in upper or lower airways were scored in all horses (Additional File 2) and video endoscopy was recorded for re-evaluation. Bronchoalveolar lavage (BAL) of the left lung was performed in all horses. After local splash anaesthesia of the trachea and bronchi with 15–20 mL lidocaine (Lidocainhydrochlorid 2%®, bela-pharm GmbH, Vechta, Germany), the endoscope was wedged in a randomly chosen bronchus. A bolus of 250 mL pre-warmed phosphate-buffered saline (PBS; Dulbecco’s Phosphate Buffered Saline, Sigma-Aldrich, Missouri, USA) was instilled and immediately regained with a low flow suction pump into a sterile silicone-coated glass bottle and checked visually for quality (white and foamy character). This step was repeated with another 250 mL PBS at the same location. Both fractions of bronchoalveolar lavage fluid (BALF) were saved separately and termed BALF 1 and BALF 2, respectively. Horses were kept in the clinic for a minimum of 24 h afterwards and clinically re-examined before discharge. Some of the horses stayed in the clinic for other diagnostic procedures or treatment.

Bronchoalveolar lavage fluid was processed within 60 min after endoscopy in-house via cytospin preparation technique for quantitative cytological analysis. Therefore, 500 µL of each BALF fraction were used. Samples were stained with Pappenheim's staining and 200 cells were counted from every BALF fraction (= 400 cells/horse). Microscopy was performed by a trained medical technician or veterinarian in × 1000 magnification with oil (Laborlux 12, Type 020-435.025, Ernst Leitz Wetzlar GmbH, Germany). In case of suspicious samples (e.g., small number of cells, damaged cells) a lower number of cells was counted, and the sample re-evaluated by a second evaluator. Cytology results were averaged afterwards. Cut-off values for BALF cytology for clinical diagnoses were < 10% neutrophils, < 2% eosinophils and < 5% mast cells for healthy horses; > 10% neutrophils, and/or > 2% eosinophils and/or > 5% mast cells for mild and moderate EA and > 25% neutrophils for severe equine asthma. Cut-offs were determined before classification of the horses and BALF cytology was used as main classification parameter, since it is an objective and quantitative parameter. Clinical signs were evaluated as absent or present for grouping of the horses but signs not evaluated for severity in this classification, which means that e.g., elevated breathing effort at rest was not used as classification for sEA per se, as stated in the ACVIM Consensus Statement [1].

### Statistical analysis

Statistical analysis was performed with the programming software R version 4.3.0 [18]. Correlations were described with Spearman’s ρ. Diagnoses were further processed as ranked variables under the assumption that severity increases from healthy to sEA.

The ‘FactoMineR’ R-package [19] was used to perform factor analysis of mixed data (FAMD) on all scaled quantitative and qualitative variables, except for the diagnosis. This dimensionality reduction technique can be used to represent complex datasets on fewer dimensions called principal components, which aggregate the data’s most variable features. The coordinates of each individual on a plot relate to their similarity. By colouring the individuals by diagnosis (which was not included in the FAMD), the relationship between this variable and the rest of the dataset becomes apparent.

The most discriminative variables regarding diagnosis were identified among all or subsets of the parameters recorded with each type of examination using recursive partitioning trees as implemented in the ‘rpart’ R-package [20]. The ‘rpart’ function was given the argument ‘minsplit = 5’ to accommodate the relatively small group sizes. The resulting classification trees can be interpreted as decision trees to reach a diagnosis from the selected examination results at given cut-offs. Scores were analysed using Kruskal–Wallis tests with Dunn’s post-hoc comparisons. Arterial blood gases were analysed using one-way ANOVA with Tukey or Dunnett’s post-hoc tests. *P*-values were adjusted for multiple comparisons with the Holm procedure following the omnibus test. Significance was set to *P* ≤ 0.05.

## Results

### Study population and medical history

Demographics of the overall population are presented in Table 2; the questionnaire is presented in Additional File 1.

**Table 2:** Demographics and medical history obtained from the owners through a standardised questionnaire

Parameter	Value
*Sex*	
Gelding	11
Mare	15
*Age*	
Mean (± SD)	11.3 (± 3.54)
Median [Min, Max]	10.8 [4.0, 18.8]
*Housing*	
Open barn or free access to paddock	13
Box stall with window	10
Box stall without window	3
*Bedding*	
Straw	17
No straw (e.g., shavings)	9
*Roughage type*	
Dry hay	18
Haylage	3
Soaked hay	2
Steamed hay	3
*Clinical signs noticed by owners*	
Cough	12
Nasal discharge	13
Dyspnoea	7
Exercise intolerance	9

### Clinical diagnoses

**Fig. 1:**
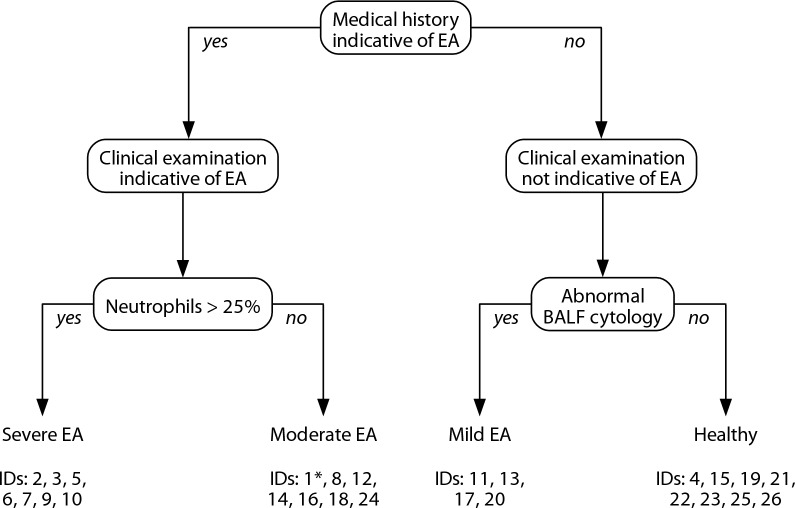
Flowchart of clinical diagnoses of all study horses. Diagnosis was based on medical history, occurrence of clinical signs at clinical examination and BALF cytology. The diagnostic algorithm was slightly deviated from in one case (ID 1), where physiological arterial blood gas results and inconspicuous clinical examination supported an overall diagnosis of ‘moderate EA’ despite a history of coughing and BALF neutrophilia of 25.5%. (Abbr.: EA = equine asthma, BALF = bronchoalveolar lavage fluid)

Clinical diagnoses were primary based on amount of mast cells, neutrophils and eosinophils in BALF cytology. History and clinical signs for respiratory disease were additionally considered as absent or present but severity not used for grouping of the horses. Horses were grouped by one veterinarian as presented in Fig. 1, in which the examination steps are shown in chronological order and horse IDs of each group are presented. However, three out of 26 horses did not fit clearly into a diagnosis by this classification tree. In one horse (ID 1) with a history of cough but no clinical signs at examination, BALF cytology revealed severe airway neutrophilia (25.5%). In this case, the clinical diagnosis of modEA was supported by physiological arterial blood gas analysis results. Furthermore, slightly elevated breathing effort at rest in two horses (ID 4 and 13) was rated as non-pathological due to behavioural reasons (ID 13), stress at examination (ID 4) and missing of additional pathological findings (both). A respiratory rate of 20/min in ID 4 was accepted due to breed (Icelandic horse) and stress in this horse. Radiographical images were screened to exclude horses with abnormalities pathognomonic for other diseases, such as intra thoracal masses or shadowing suspicious for equine multinodular lung fibrosis. None of these were found in any of the horses. The mean age (± SD) was 11.0 (± 4.15) years for healthy horses, 8.28 (± 1.78) for miEA, 12.4 (± 3.73) for modEA and 12.2 (± 2.79) for sEA.

### Associations between clinical diagnosis and examination parameters

**Fig. 2:**
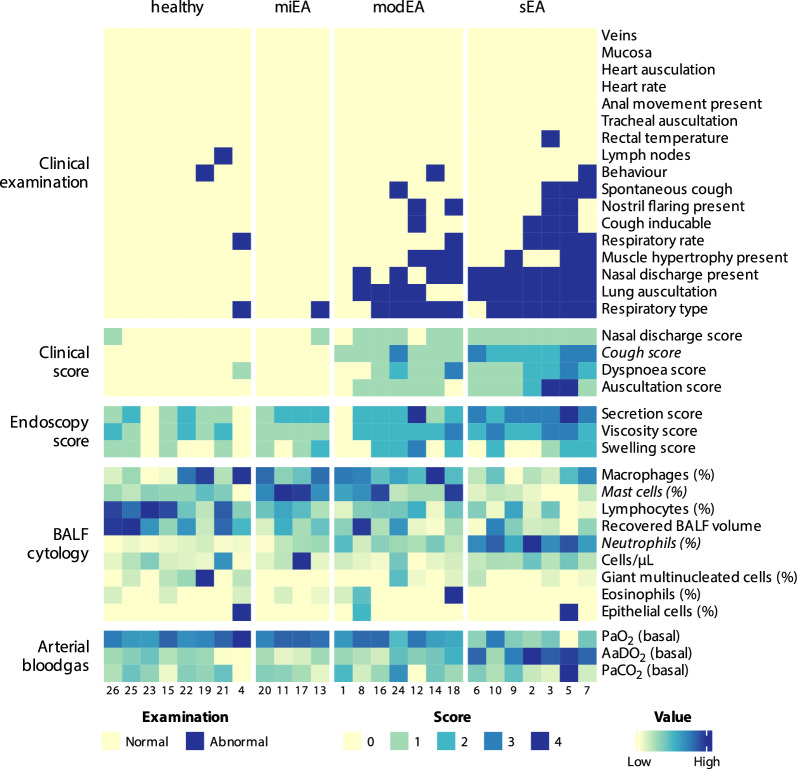
Heatmap of the main results from the standardised examination. Each column represents one horse and each row represents one parameter of the protocol. The clinical diagnoses are reflected in patterns in different sections of the heatmap. Prevalence of abnormal clinical findings and elevated scores are associated with disease severity. Similarly, percentage of neutrophils in BALF and AaDO_2_ are higher in affected horses, while the opposite is true for lymphocytes. Detailed results from all examinations can be found as Additional Files 2–5. Parameters selected in the algorithmic re-evaluation of diagnoses (Fig. 5) are presented in italics (neutrophils, mast cells, cough score). (Abbr.: EA = equine asthma, miEA = mild EA, modEA = moderate EA, sEA = severe EA, BALF = bronchoalveolar lavage fluid, PaO_2_ = arterial oxygen partial pressure, AaDO_2_ = Alveolar-arterial oxygen gradient, PaCO_2_ = arterial carbon dioxide partial pressure)

There were no single examination results that sufficiently differentiated defined EA phenotypes from one another. A lack of clinical signs in healthy and miEA horses was predetermined by the classification strategy. Since mainly defined by BALF cytology, the clinical presentations of horses in modEA and sEA were diverse. An overview of all examination results is visualised in Fig. 2. Respiratory symptoms were more frequent in sEA than modEA (clinical score: modEA = median 4; range 6 and sEA = median 7; range 6). Nevertheless, the clinical picture was not consistent in all horses of one phenotype and there is some overlap among scores within groups (modEA scores 1–7; sEA scores 5–11). Dyspnoea at rest was not always associated with severe airway neutrophilia and was therefore also present in some horses classified as modEA (e.g., ID 18). Detailed results of the examinations can be found in Additional Files 3–6.

**Fig. 3:**
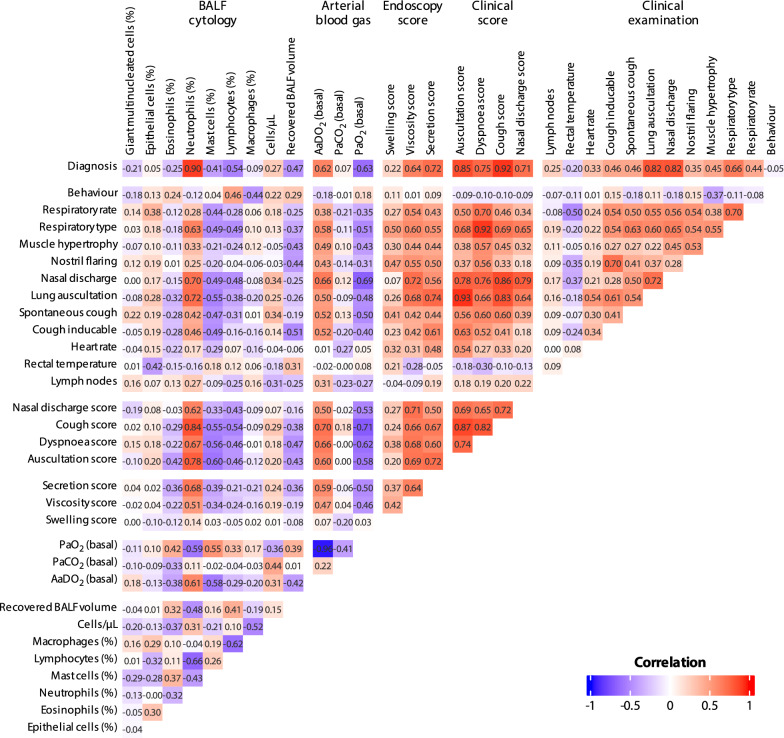
Correlation matrix with Spearman’s correlation coefficients for all variables. As some of the variables are not independent of the diagnosis (healthy, mild EA, moderate EA, severe EA), the correlations must be interpreted accordingly. The cough score, as part of the clinical score, showed the strongest correlation with diagnosis, highlighting the importance of medical history in equine asthma. (Abbr.: EA = equine asthma, BALF = bronchoalveolar lavage fluid, PaO_2_ = arterial oxygen partial pressure, AaDO_2_ = Alveolar-arterial oxygen gradient, PaCO_2_ = arterial carbon dioxide partial pressure)

Spearman’s correlation coefficients for all variables of the examinations are presented in Fig. 3. Correlations represent trends of the importance and associations of examination parameters taken in this study and need to be interpreted with the knowledge of the ranked phenotype definitions as presented above. Of all parameters from the clinical examination, nasal discharge and lung auscultation had the strongest association with disease severity (ρ = 0.82, *P* < 0.001). However, clinical scoring appeared superior to the underlying single subjective parameters regarding their association with disease severity, which emphasizes the benefit of using more objectively defined diagnostic scores (ρ = 0.75–0.92, *P* < 0.001). The correlations between single subjective parameters and the corresponding scores (e.g., subjective lung auscultation and auscultation score (ρ = 0.93, *P* < 0.001)) are generally strong.

There were no anatomical or pathological abnormalities of the upper airways at endoscopic examination, apart from minor sedation-induced asymmetry of the arytenoid cartilage. The overall endoscopy score was significantly different between groups (see Table 3). Group medians and ranges were 3.5 (4.0) for healthy horses, 3.5 (2.0) for miEA, 6.0 (9.0) for modEA and 7.0 (4.0) for sEA. However, ranges of the subscores were large (Additional File 4). Mucus accumulation in the trachea, described as secretion score, showed the strongest association to disease severity (ρ = 0.72, *P* < 0.001) and clinical parameters such as lung auscultation (ρ = 0.74, *P* < 0.001). However, endoscopy scores were not superiorly associated with the disease severity compared to the clinical scoring as indicated by higher *P*-values and less significant post-hoc comparisons shown in Table 3.

**Table 3:** Clinical and endoscopic subscores are insufficient to differentiate between equine asthma phenotypes and healthy horses

Parameter	Test statistic (H)	ε^2^ [95% CI]	*P*-value	Adj. *P*-value	Healthy	miEA	modEA	sEA
Nasal Discharge Score	13.2	0.53 [0.35–1]	0.004	0.017	a	ab	ab	b
Cough Score	23.1	0.93 [0.88–1]	0	0	a	ab	bc	c
Dyspnoea Score	15.8	0.63 [0.51–1]	0.001	0.007	a	a	ab	b
Auscultation Score	19.6	0.79 [0.74–1]	0	0.001	a	a	ab	b
Clinical Score	21	0.84 [0.8–1]	0	0.001	a	ab	bc	c
Secretion Score	13.8	0.55 [0.44–1]	0.003	0.016	a	ab	ab	b
Viscosity Score	12.2	0.49 [0.38–1]	0.007	0.02	a	a	ab	b
Swelling Score	2.18	0.09 [0.04–1]	0.535	0.535	–	–	–	–
Endoscopy Score	11.8	0.47 [0.4–1]	0.008	0.02	a	ab	ab	b

Overview of score comparisons between diagnoses using Kruskal–Wallis tests. The results indicate that the clinical use of the presented subscores is limited due to overlapping scores among phenotypes. The Kruskal–Wallis test statistics (H) and the effect sizes epsilon squared (ε^2^) are presented, alongside the latter’s 95% confidence intervals. All scores but the swelling score show significant differences between the groups (adj. *P*-values ≤ 0.05), which indicates overall large variations between healthy and diseased animals. Different letters in the ‘healthy’, ‘miEA’, ‘modEA’ and ‘sEA’ indicate significant differences between those groups in Dunn’s post-hoc comparisons. For example, the endoscopy score significantly differs between the healthy and sEA group (letters a vs. b), but neither miEA nor modEA are significantly different from the other groups, as they share the letters a, b, or both. It follows that the significant results from the Kruskal–Wallis tests are mainly attributable to the sEA group, which shows the most marked differences compared to the remaining phenotypes. All horses were included in the analysis (*n* = 26). The *P*-values from the Kruskal–Wallis tests were adjusted for multiple comparisons with the Holm procedure. (Abbr.: Adj. *P*-value = adjusted *P*-value, miEA = mild equine asthma, modEA = moderate equine asthma, sEA = severe equine asthma)

The total amount of recovered BALF was negatively correlated with the severity of the disease (ρ = −0.47, *P* = 0.016; group means ± SD were for healthy 323.75 mL (± 67.58), for miEA 251.25 mL (± 38.79), for modEA 258.57 mL (± 75.05) and for sEA 231.42 mL (± 55.78) or in total 64.75, 50.25, 51.71 and 46.28%, respectively). From the BALF samples, 400 cells were counted per horse, expect from ID 14, in which the sample of BALF 1 was not sufficient and 300 cells were counted in BALF 2. BALF 1 contained on average less cells/µL than BALF 2 (overall means 113.5vs. 155.80 cells/µL). Higher percentages of neutrophils were found in BALF 1 (overall means 22.2 vs. 13.8%), whereas macrophages and lymphocytes where higher in BALF 2 (overall means 32.1 vs. 37.4% and 40.8 vs. 43.3%). All horses grouped as miEA showed a mastocytic cell infiltration of the lung (4/4), three out of four horses additionally showed an elevated neutrophil count. In modEA, only four out of seven horses had a mastocytic involvement in addition to elevated neutrophil counts. Horses with severe EA did not show elevations of other cell types than neutrophils. Spearman’s correlations of diagnosis and BALF results must be interpreted with caution, since neutrophils (ρ = 0.90, *P* = 0.001) and mast cells (ρ = −0.41, *P* = 0.039) were used to define the diagnosis. Interestingly, giant multinucleated cells were negatively correlated with disease severity, since they were only found in healthy horses in this study (ρ = −0.21, *P* = 0.011).

**Fig. 4:**
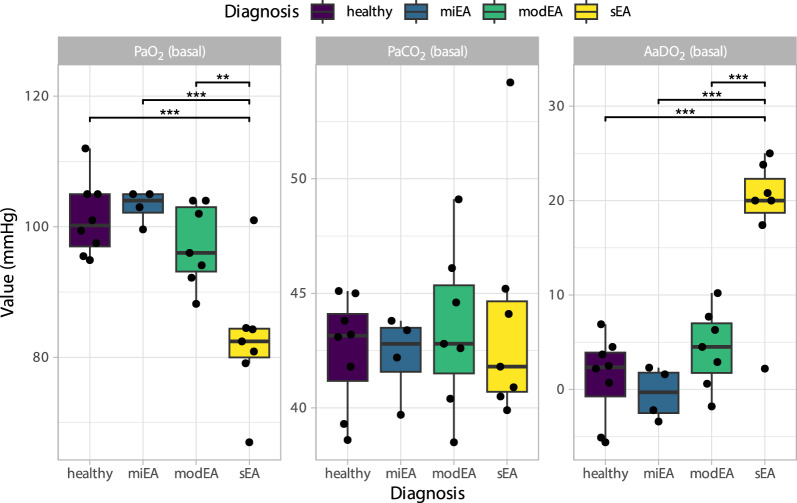
Arterial blood gas analysis at rest. PaO_2_ and AaDO_2_ were significantly different between groups (PaO_2_ F (3, 22) = 11.2, ω^2^_p_ = 0.54 [0.25–1], adj. *P* < 0.001; and AaDO_2_; F (3, 22) = 17.3, ω^2^_p_ = 0.65 [0.41–1], adj. *P* < 0.001). However, this was only attributable to the sEA group, differing from all others (adj. *P* ** = 0.001; *** < 0.001). PaCO_2_ was comparable between the groups (F (3, 22) = 0.265, ω^2^_p_ = 0 [0–1], adj. *P* = 0.85). (Abbr.: EA = equine asthma, miEA = mild EA, modEA = moderate EA, sEA = severe EA, PaO_2_ = arterial oxygen partial pressure, PaCO_2_ = arterial carbon dioxide partial pressure, AaDO_2_ = Alveolar-arterial oxygen gradient)

Results from the arterial blood gas analysis are presented in Fig. 4. The partial arterial oxygen pressure (PaO_2_) was significantly different between groups (F (3, 22) = 11.2, ω^2^_p_ (partial omega-squared) = 0.54 [0.25–1], adjusted (adj.) *P* < 0.001), however, pairwise comparisons only revealed a significant decrease in sEA compared to all other groups (*P* = 0.006–0.031). Similarly, the alveolar-arterial oxygen gradient (AaDO_2_; F (3, 22) = 17.3, ω^2^_p_ = 0.65 [0.41–1], adj. *P* < 0.001) only differed between severely affected horses and all other groups. In contrast, the partial arterial carbon dioxide pressure (PaCO_2_) did not show significant differences between the groups (F (3, 22) = 0.265, ω^2^_p_ = 0 [0–1], adj. *P* = 0.85). The alveolar-arterial oxygen gradient (AaDO_2_) was calculated from PaO_2_ and PaCO_2_ results and showed significant differences between the groups. Interestingly, the arterial oxygen partial pressure and the alveolar-arterial oxygen gradient showed inversely comparable associations to all tested parameters of the used data subsets. For group means and individual values see Additional File 5.

All blood parameters, such as haematology including differential blood count, total plasma protein, electrolytes and fibrinogen were within reference values in all horses. Group comparisons showed no significant group differences except for post-hoc analysis of total plasma protein between healthy and sEA (adj. *P* = 0.02). Dunnett’s test also revealed non-significantly elevated fibrinogen levels in sEA compared to healthy horses (adj. *P* = 0.0591) and a trend of lower eosinophil counts with disease severity (healthy vs. sEA, adj. *P* = 0.0777). Graphs are presented in Additional File 6.

### Algorithmic re-evaluation of the diagnostic decision tree

**Fig. 5:**
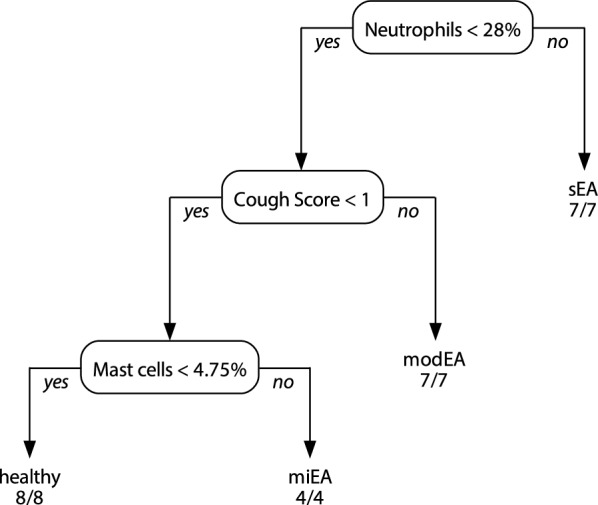
Decision tree showing the variables selected among all available data by a recursive partitioning tree. The presented tree selected the variables ‘Cough score’, ‘Mast cells in BALF’ and ‘Neutrophils in BALF' from the whole data with automatically identified cut-offs to attain the clinical diagnoses with 100% accuracy. (Abbr.: EA = equine asthma, BALF = bronchoalveolar lavage fluid, miEA = mild EA, modEA = moderate EA, sEA = severe EA)

Figure 5 presents a decision tree obtained from a recursive partitioning algorithm used on all data, which is an objective technique to re-evaluate needed data subsets for group assignments. The algorithm automatically selected variables and cut-offs to predict the before classified groups presented as diagnoses with a 100% accuracy. Only the three variables ‘BALF neutrophils’, ‘BALF mast cells’ and the ‘cough score’ were needed to obtain the same assignment to phenotypes in all horses. The cut-offs were not identical to the ones used to determine the clinical diagnosis, as neutrophils are presented with a cut-off of 28% and mast cells distinguish healthy and miEA horses at 4.75%.

### Diagnostic potential of subsets of variables

**Fig. 6:**
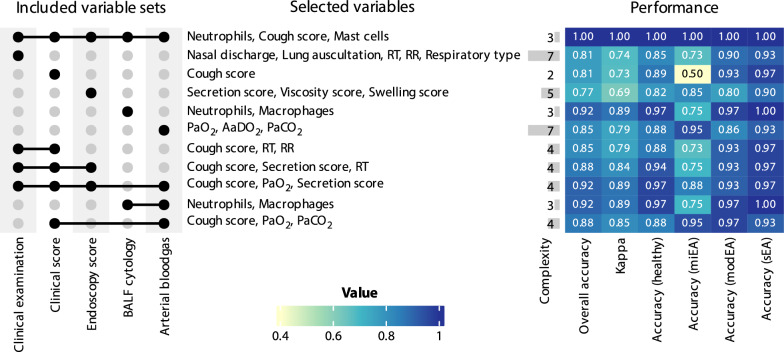
Performance of classification trees to distinguish phenotypes. Different subsets of the overall examination data were used. Each row corresponds a classification tree based on different subsets of data. The left part of the figure indicates which subsets of variables were available to the model (black dots). The central part shows which variables were retained in the decision tree, whose complexity is indicated by its number of nodes. The first line corresponds to the tree, which has only 3 nodes and retained the three variables ‘cough score’, ‘BALF neutrophils’ and ‘BALF mast cells’. Some trees have up to 7 nodes, which is indicative of overfitting, making these trees useless in a realistic setting. Finally, the trees performance is shown as heatmap in the right. This analysis highlights the critical importance of BALF cytology in the differentiation of the EA phenotypes. Furthermore, it indicates that the diagnosis of miEA as defined in this study is the most challenging with the selected examinations and requires BALF cytology. (Abbr.: BALF = bronchoalveolar lavage fluid, EA = equine asthma, miEA = mild EA, modEA = moderate EA, sEA = severe EA, RR = respiratory rate, RT = rectal temperature, PaO_2_ = arterial oxygen partial pressure, PaCO_2_ = arterial carbon dioxide partial pressure, AaDO_2_ = Alveolar-arterial oxygen gradient)

All examination data subsets were analysed for their benefit in diagnosing the ranked EA phenotypes in this study. This approach helps to objectively evaluate large amounts of data in one statistical model. Therefore, several other classification trees were generated using subsets of variables instead of the whole data set, to quantify the diagnostic potential of each examination step. As indicated in Fig. 6, no combination of examination steps was superior to the full model which relied on neutrophils, mast cells and the cough score (algorithmic classification shown in Fig. 5).

### Overall phenotypical presentation of the study population

**Fig. 7:**
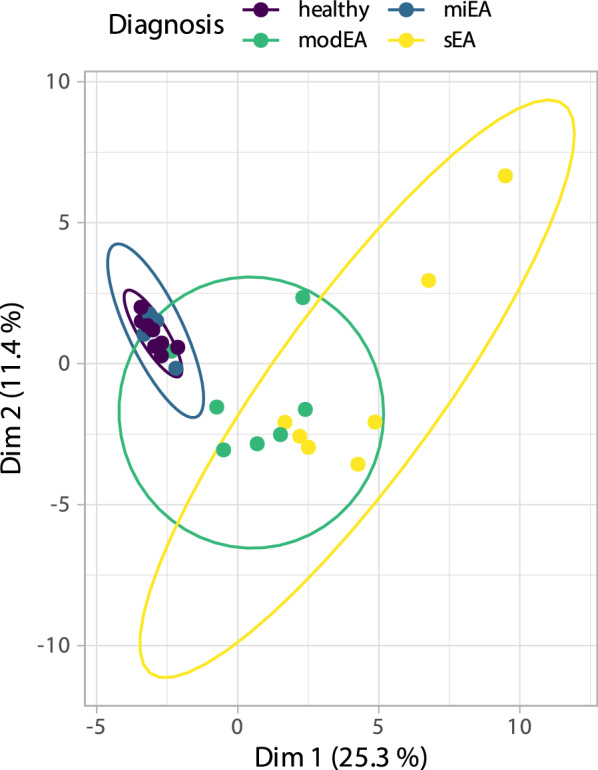
Factor analysis of mixed data on the whole data set. The clinical presentation of the three EA phenotypes in comparison to lung healthy controls are visualised. Every point embodies a single horse. The colours indicate the diagnosis, which was not included in the analysis. A 68% confidence ellipse is shown for each group. Healthy and subclinical diseased horses (miEA) share almost the same presentation. The heterogeneity of the clinical presentation increases with disease severity from healthy to sEA. However, ranges of the three phenotypes are overlapping, which highlights the complexity of the clinical presentations of EA phenotypes. (Abbr.: Dim = dimension, EA = equine asthma, miEA = mild EA, modEA = moderate EA, sEA = severe EA)

Factor analysis of mixed data was performed on the whole data set (except for the diagnosis) to provide a global representation of the phenotypic variation in the study population (Fig. 7). The resulting plot summarises about 37% of the data set’s total variance and reflects the greater variability of more pronounced phenotypes. In contrast, the miEA group clusters together with the healthy horses.

## Discussion

The present study consisted in the description of the phenotype variability associated with EA in comparison to healthy controls in a cohort of horses in Germany. The horses were grouped as mildly, moderately and severely affected (miEA, modEA, sEA) by BALF cytology and occurrence of any clinical sign in accordance with respiratory disease. Cough score and BALF cytology were confirmed as accurate predictors of the defined EA phenotypes by use of recursive partitioning trees, which did not identify sufficiently informative alternative variables in all examination data.

### Diagnostic value of specific examinations

In practice, clinical examination and BALF cytology are essential in the diagnosis of EA [1]. However, the variability of EA phenotypes can justify a broad range of examinations for full disease characterisation. The dynamic nature of EA and lack of temporal consistency in patients exposed to changing environments emphasizes the importance of owner observations and results in clinical variability [21–23].

From medical history and clinical examination, the cough score was identified as a potent discriminant parameter in the classification tree in this study. Some authors suggest that a diagnosis of sEA can be made based solely on medical history [8]. However, it has been shown that owners have difficulty in the recognition and assessment of EA [24]. Previous studies have evaluated the reliability of owner observations in describing the severity of RAO. These studies have found reliable agreement of owner reports with severe symptoms of RAO [25–27] and a good association of owner-reported cough and clinical signs in affected horses [28]. The present study confirms this finding, as cough was the only owner-reported symptom that reliably distinguished between healthy and clinically diseased horses. The cough score used in this study included both cough at examination and history of coughing. It is therefore not an exact clinical score; however, the authors selected this description to highlight the potential applicability in a field setting with anamnesis and clinical variables. Analysis revealed that the combination of anamnesis and clinical examination is advantageous to single parameters alone. Still, it is important to note that reported cough as indicator is only applicable to modEA and sEA, is influenced by frequency of observations and is of no benefit in diagnosing subclinical EA (mild = miEA).

Airway endoscopy with BALF collection is considered the gold standard of EA diagnosis [1, 21, 29]. In this study, classification trees without BALF data were unable to achieve clinically realistic complexities with sufficient performance accuracies, regardless of the data subsets used. This highlights the significant importance of BALF examination. There are discussions about feasible cut-off values for equine asthma [1], which have resulted in various cut-offs being used in different studies [4, 21, 27, 30]. In this study, the cut-offs of 10% for neutrophils, 5% for mast cells and 2% for eosinophils were chosen to avoid over-diagnosing EA in horses examined at different seasons of the year or being fed different roughage types before presentation to the clinic, since these factors can influence inflammatory cell influx [4, 31]. The classification algorithm suggests cut-off values of 4.75% for mast cells to differentiate healthy from miEA and 28% for neutrophils to differentiate modEA from sEA. These cut-offs are specific for the presented study population and should not be generalized to a population level. Nevertheless, investigated under the assumption of ranked severity of diagnoses, it emphasizes the involvement of mast cells in early detected EA and the importance of airway neutrophilia for disease severity in this cohort of horses in Germany.

Endoscopic scores were increasing with disease severity but overlapped between the phenotypes. These findings are consistent with previous studies that have shown little value of single visual abnormalities at endoscopy for diagnostics alone [6, 32–34]. Two studies found good discriminant potential in the amount of tracheobronchial mucus between control and RAO-affected horses [35, 36]. In our study, the amount of tracheobronchial mucus (secretion score) had the strongest positive association with disease severity and BALF neutrophils. Additionally, it was found that the recovered BALF volume was negatively correlated with disease severity. This phenomenon may be attributed to airway remodelling, increased alveolar spaces or lung emphysema, decreased compliance or bronchospasm in sEA horses [37–39]. However, all these findings did also not distinguish miEA and modEA from healthy horses well.

Arterial blood gas analysis has been suggested as an aid in scoring EA severity [29, 40, 41]. In this study, PaO_2_ was in fact lower in the sEA group but stable in less severely affected horses. The decision tree based on arterial blood gas parameters was overly complex and performed poorly to separate all horses into their respective phenotypes. Moreover, other models where this data was available did not select these parameters. In conclusion, physiological arterial blood gas values cannot rule out any EA phenotype, since physiological values were also present in one horse classified as sEA. Still, a decreased PaO_2_ were found in most cases of sEA and underline the severity of the disease.

There are difficulties in the objective evaluation of lung radiographs, such as the influence of body condition score or breed and were shown to have low predictive value in mEA [42]. Radiographs were therefore not evaluated in regard of EA severity. However, they aided in excluding diseases such as EMPF, thoracal masses or severe interstitial lung disease and were therefore beneficial.

### Phenotypical variability in a clinical population

A major finding of the present study was the association between EA severity and clinical phenotype variability. Healthy and miEA horses share the similar inconspicuous clinical presentation and can only be distinguished by BALF cytology, since these phenotypes were defined by these two factors. In each horse diagnosed with modEA and sEA at least one pathological finding was noticeable during the clinical examination. However, the frequency and severity of clinical signs did not increase with assigned diagnosis in all individuals per phenotype, as indicated by overlapping scores. The clinical scores correlated well with the underlying parameters of the clinical examination and were also superiorly associated with disease severity, supporting the use of defined scores as objective variables. According to our phenotype definitions, elevated respiratory effort at rest was widely distributed and not exclusively assigned to the sEA phenotype, as suggested by the ACVIM consensus definition [1]. In our definition, the absence of severe clinical signs did not preclude severe airway inflammation (e.g., ID 10 and 7) and vice versa (e.g., ID 18). The wide variety of clinical signs in mEA, including elevated breathing effort, was previously described in other asthmatic horse populations [4, 27] and needs further evaluation. In this study, the frequency and severity of additional symptoms next to elevated breathing effort varied among symptomatic horses of both phenotypes. Spontaneous cough at hospitalisation was present in some horses with modEA and sEA; and lung auscultation was also found to be physiological in horses classified as sEA. The insensitivity of lung auscultation in mild and modEA cases has been recently objectively confirmed by using a digital auscultation device [43] and other authors suggested that lung auscultation is not beneficial for EA classification [35]. Additionally, nasal discharge was rather consistent among symptomatic horses (11/14), although it is not emphasized in the definition of EA [1, 7]. Another study reported this symptom in approximately 40% of horses with mEA and sEA [27]. We found associations between the occurrence of symptoms and probability for advanced disease in all clinical examination findings and clinical scores. However, the discriminatory potential of each parameter was poor, and the absence of individual symptoms did not necessarily indicate the absence of severe disease. Management and housing before presentation may explain the variability of the phenotypes. Antigen avoidance was shown to have a high impact on the clinical presentation, possibly also influencing measurable airway neutrophilia and remodelling [5, 21–23, 44–48]. In some horses, clinical signs improve before resolution of BALF neutrophilia [49]. Together, these findings indicate that repeated examinations may be ideal to correctly classify a horse, as short-term changes may affect clinical parameters before affecting BALF cytology. The overlapping clinical presentations of the phenotypes and the increasing clinical variability of all examinations were confirmed by factor analysis of mixed data, where increasingly large clusters were observed for each group.

### Supporting the existence of a mild EA phenotype

The 2016 ACVIM Consensus Statement distinguishes between two phenotypes (mEA and sEA). The mEA definition groups subclinically and mildly diseased horses as a single phenotype, while also acknowledging that EA is a spectrum [1]. However, the presence of clinically inconspicuous horses with abnormal BALF cytology is a challenge. In this study, four out of 12 as healthy presented horses showed signs of airway inflammation in BALF cytology. The definition of the subclinical phenotype as mild EA was suggested for racehorses with abnormal BALF findings and exercise intolerance [5, 10, 50]. However, populations of subclinical non-racehorses with abnormal BALF cytology were not just described in our study, which suggests the use of this phenotype description also for other horse populations than equine athletes [4, 6, 8]. Diagnosing subclinical EA more frequently may aid to reduce the prevalence of symptomatic horses by emphasizing to horse owners, the need for antigen control, also for clinically healthy horses. This is under the assumption of subclinical disease progressing to clinical EA. However, diagnosing miEA is difficult because low-level exercise intolerance is much harder to define and detect in leisure horses, due to the variety of disciplines, expected and expectable performance [51–53]. In addition, standardised treadmill tests are not available under field conditions [54–56]. Finally, portable devices for pulmonary function testing and blood biomarkers are missing, and bronchoprovocation tests do not seem reliable in miEA [57–59]. Consequently, BALF cytology remains the only applicable diagnostic method for subclinical horses that can also be performed under field conditions. However, the clinical relevance of slightly elevated inflammatory cells in BALF in otherwise inconspicuous horses is insufficiently supported by literature [8, 21]. Lung function testing was not available in this study and might have helped to further elucidate this point. Since BALF cytology is not performed on a regular basis in horses with subclinical EA, this phenotype might be underdiagnosed in the German horse population [8]. However, due to a small group size, the results must not be overinterpreted on a population level.

### Main limitations

The standardised examination protocol was advertised through an online campaign to horse owners. Therefore, the study population is likely to be biased towards affected horses and pre-informed owners. However, the aim of the study was to describe the breadth of EA phenotypes rather than prevalence of each phenotype. Moreover, the predefined protocol ensured objective examination results and consistent testing conditions. The use of a single examiner may be seen as a limitation, but this approach was taken to avoid examiner variance.

Another issue affecting EA diagnostics in general is the difficulty of defining and assessing exercise intolerance, which was mentioned in the medical history by some owners, but could not be identified as an objective, repeatedly detectable and quantifiable symptom. As a result, low-level exercise intolerance cannot be excluded with the underlying data.

Furthermore, the diagnoses were ranked from miEA to sEA for analysis. It is not yet known whether miEA progresses to modEA and sEA, and this must be taken into account when interpreting the results of the study.

Finally, the study population is limited in size and horses in the mild EA group decreased, since clinically inconspicuous horses are rarely presented for BALF cytology in a clinical setting. Therefore, the decision trees are expected to be less performant when used on a broader population. Since the main tree involves only three variables (cough score, mast cells, neutrophils) and is appealing by its simplicity, a validation study on a separate cohort is warranted.

## Conclusions

In this study, medical history, clinical examination, endoscopy, BALF cytology and arterial blood gas findings were described and compared among horses exhibiting three EA phenotypes and healthy individuals. BALF cytology was found to be essential to identify subclinical cases, characterized by elevated mast cell counts (algorithmic cut-off of 4.75% in this population). Among symptomatic horses, best identified using a cough score, modEA and sEA were differentiated by neutrophil counts (algorithmic cut-off of 28% in this population). No other sets of clinical variables were comparably performant to reach an accurate diagnosis. The overall clinical variability of the phenotypes was shown to increase with disease severity, if defined mainly by BALF cytology. While the presence of several symptoms reliably indicated EA, the absence of single abnormalities did not exclude advanced disease. Subclinical disease as defined in this study may be detected earlier by use of BALF cytology in unsuspected horses but the clinical relevance of mild pathological BALF findings needs further investigation.

## Supplementary Information

Additional file 1. Questionnaire sent to the horse owners before presenting their horse to the clinic (translated from German).

Additional file 2. Scoring system used for endoscopic examination of the lower respiratory tract within the clinic as published by Fey and Venner, 2017. Scores from 0-5 for the accumulation of mucus were partly adapted from Gerber et al, 2004.

Additional file 3. Results from the clinical examination and clinical scoring of the individual horses grouped in diagnoses. (Abbr.: EA=equine asthma, RR=respiratory rate).

Additional file 4. Endoscopy and BALF cytology results. Horses and results are grouped according to their diagnoses. A total of 400 cells were counted per horse, expect from ID 14, in which the sample of BALF 1 was not sufficient and 300 cells were counted in BALF 2. The results from BALF 1 and 2 were pooled to obtain the results displayed in this table. (Abbr.: EA=equine asthma, BALF=bronchoalveolar lavage fluid, Macro=macrophages, Lymph=lymphocytes, MC=mast cells, Eos=eosinophils, GMC=giant multinucleated cells).

Additional file 5. Arterial blood gas analysis. Results are sorted by ID as horses were presented to the clinic. The arterial partial pressure of O2 and CO2 were measured and the alveolar-arterial oxygen gradient was calculated in all horses. The mean group values for PaO2 were 101.79 mmHg (± 5.93) for healthy horses, 103.15 mmHg (± 2.55) for miEA, 97.21 mmHg (± 6.23) for modEA, and 82.75 mmHg (± 10.03) for sEA. The group means for PaCO2 were 42.49 mmHg (± 2.43) for healthy horses, 42.27 mmHg (± 1.84) for miEA, 43.44 mmHg (± 3.54) for modEA, and 43.80 mmHg (± 4.98) for sEA. The group means for AaD 02 were 1.2 mmHg (± 4.4) for healthy horses, -0.4 mmHg (± 2.8) for miEA, 4.3 mmHg (± 4.1) for modEA and 18.5 mmHg (± 7.6) for sEA. (Abbr.: EA=equine asthma, miEA=mild EA, modEA=moderate EA, sEA=severe EA, PaO2=arterial oxygen partial pressure, PaCO2=arterial carbon dioxide partial pressure, AaDO2=Alveolar-arterial oxygen gradient).

Additional file 6. Results from the blood examinations grouped in diagnoses. All values were in normal limits and group comparisons did not reveal any significant group differences. Dunnett’s post-hoc test of total plasma protein (measured via refractometry) showed a significant difference between healthy and sEA (adj. P=0.02), which may be attributed to the trend of higher fibrinogen levels in sEA horses (adj. P=0.0591). There was also a non-significant trend of lower eosinophils with disease severity (healthy vs. sEA, adj. P=0.0777). However, since results were all within the reference values and group sizes limited, findings do not seem to be beneficial for clinical diagnostics. (Abbr.: Adj. P = adjusted P-value, EA=equine asthma, sEA=severe EA).

## Data Availability

The datasets used and analysed during the current study are available from the corresponding author on reasonable request.

## Abbreviations

ACVIM: American College of Veterinary Internal Medicine
BAL: Bronchoalveolar lavage
BALF: Bronchoalveolar lavage fluid
EA: Equine asthma
EMPF: Equine multinodular pulmonary fibrosis
IAD: Inflammatory airway disease
mEA: Mild-moderate EA
miEA: Mild EA
modEA: Moderate EA
PBS: Phosphate-buffered saline
RAO: Recurrent airway obstruction
RR: Respiratory rate
sEA: Severe EA
