# Supplementary material for: Clinical variability of equine asthma phenotypes and analysis of diagnostic steps in phenotype differentiation
Source: Acta Vet Scand. 2024 Sep 18;66:51. doi: 10.1186/s13028-024-00773-7 (PMC11409572; doi:10.1186/s13028-024-00773-7)
Supplement: Supplementary file 1 — Additional file 1. Questionnaire sent to the horse owners before presenting their horse to the clinic (translated from German). [file 13028_2024_773_MOESM1_ESM.pdf]

Additional File 1 (PDF): Questionnaire sent to the horse owners before presenting their horse to the clinic (translated from German).

1. When did you buy your horse?
2. How do you keep your horse? (Free text: please indicate type of housing, type of bedding and hours per day in pasture or paddock)
3. What type of forage/roughage do you feed your horse (dry hay, steamed hay, washed hay, haylage, other: please specify)? How do you feed the roughage (from the ground, hay net, hay rack, other: please specify)?
4. Do you know of any chronic illness (e.g., PPID, EMS, asthma)? If wo, when was it diagnosed?
5. Is your horse on any medication? If so, what type, frequency and dosage?
6. Have you noticed any of the following symptoms in the last few months (please tick)?

Symptoms	Comment	Answer
Cough	Never	
	Sometimes (single cough every few days)	
	Single cough regularly every day or always at the start of exercise	
	Frequent daily cough or cough attacks	
Exercise intolerance	No	
	Yes	
Breathing at rest	Normal	
	At the end of exhalation, the abdomen sometimes lifts slightly	
	The abdomen rises clearly with each breath	
	Breathing and abdominal lifting appear strained and/or nostrils are wide open when breathing	
	Severe respiratory effort and reduced behaviour, severe abdominal lifting, nostril flaring, unwillingness to move	
Nasal discharge	Yes	
	No	

7. How long have you noticed individual symptoms?
8. Have you noticed an acute worsening of symptoms in the past? When did this happen? Did your horse fully recover to the health condition prior to the worsening?
9. What type of respiratory system diagnostic procedures were performed in the past?
10. What type of treatment has your horse received for lung disease in the past? When and for how long were the treatments given? How did your horse respond? Did you notice any improvement in symptoms?
11. How do you ‘use’ your horse (e.g. sport, leisure)? How many times a week do you work your horse? Have you noticed any intolerance or reduction in exercise due to lung disease? If so, when did you notice these changes?
