# Supplementary material for: Clinical variability of equine asthma phenotypes and analysis of diagnostic steps in phenotype differentiation
Source: Acta Vet Scand. 2024 Sep 18;66:51. doi: 10.1186/s13028-024-00773-7 (PMC11409572; doi:10.1186/s13028-024-00773-7)
Supplement: Supplementary file 2 — Additional file 2. Scoring system used for endoscopic examination of the lower respiratory tract within the clinic as published by Fey and Venner, 2017. Scores from 0-5 for the accumulation of mucus were partly adapted from Gerber et al, 2004. [file 13028_2024_773_MOESM2_ESM.pdf]

Parameter	*Score*	*Comment*
Accumulation (Amount of tracheal mucus)	0	No secretion
	1	Single spots
	2	Multiple spots or thin intermittent secretion line
	3	Secretion <10% of tracheal surface or thin continuous secretion line
	4	Wide secretion line and/or mucus accumulation at flexure of trachea
	5	> half of tracheal lumen mucus-filled (discontinuously)
Viscosity	0	Physiological shining of the mucosa
	1	Serous, translucent, fluent
	2	Slowly running secretion, movable with airflow
	3	Highly viscous secretion, endoscopic trace stable, not moving with airflow
Swelling	0	None
	1	Mild swelling of tracheal bifurcation
	2	Moderate swelling of tracheal bifurcation, tracheal rings indistinctly visible
	3	Severe swelling of tracheal bifurcation and/or invisible tracheal rings
Individual score (max. 10)		

Additional File 2 (PDF): Scoring system used for endoscopic examination of the lower respiratory tract within the clinic as published by Fey and Venner [1]. Scores from 0-5 for the accumulation of mucus were partly adapted from Gerber et al. [2].

References Additional File 2:

1. Fey K, Venner M. Krankheiten des unteren Respirationstraktes. In: Brehm W, Gehlen H, Ohnesorge B, Wehrend A, editors. Handbuch Pferdepraxis (4. Aufl.). Stuttgart: Enke; 2016; p. 356-416.

2. Gerber V, Lindberg Å, Berney C, Robinson NE. Airway mucus in recurrent airway obstruction- Short-term response to environmental challenge. J Vet Intern Med. 2004;18:92-7.
