# Supplementary material for: Clinical variability of equine asthma phenotypes and analysis of diagnostic steps in phenotype differentiation
Source: Acta Vet Scand. 2024 Sep 18;66:51. doi: 10.1186/s13028-024-00773-7 (PMC11409572; doi:10.1186/s13028-024-00773-7)
Supplement: Supplementary file 3 — Additional file 3. Results from the clinical examination and clinical scoring of the individual horses grouped in diagnoses. (Abbr.: EA=equine asthma, RR=respiratory rate). [file 13028_2024_773_MOESM3_ESM.pdf]

Diagnosis	ID	RR	Cough Score	Dyspnoe Score	Auscultation Score	Nasal Discharge Score	Clinical Score (overall)	Respiratory type	Muscle hypertrophy	Nasal discharge	Lung auscultation	Spontaneous cough
Healthy (n=8)	4	20	0	1	0	0	1	Forced abdominal (mild)	No	No	Physiological	No
	15	12	0	0	0	0	0	Costo-abdominal	No	No	Physiological	No
	19	16	0	0	0	0	0	Costo-abdominal	No	No	Physiological	No
	21	16	0	0	0	0	0	Costo-abdominal	No	No	Physiological	No
	22	12	0	0	0	0	0	Costo-abdominal	No	No	Physiological	No
	23	12	0	0	0	0	0	Costo-abdominal	No	No	Physiological	No
	25	12	0	0	0	0	0	Costo-abdominal	No	No	Physiological	No
	26	12	0	0	0	1	1	Costo-abdominal	No	No	Physiological	No
mild EA (n=4)	11	12	0	0	0	0	0	Costo-abdominal	No	No	Physiological	No
	13	12	0	0	0	0	0	Forced abdominal (mild)	No	No	Physiological	No
	17	12	0	0	0	0	0	Costo-abdominal	No	No	Physiological	No
	20	16	0	0	0	0	0	Costo-abdominal	No	No	Physiological	No
moderate EA (n=7)	1	10	1	0	0	0	1	Costo-abdominal	No	No	Physiological	No
	8	16	1	1	1	1	3	Costo-abdominal	No	Yes	Pathological (moderate)	No
	12	16	1	1	1	0	3	Forced abdominal (mild)	Yes	No	Pathological (mild)	No
	14	12	1	1	1	1	4	Forced abdominal (mild)	Yes	Yes	Physiological	No
	16	12	1	1	1	1	4	Forced abdominal (mild)	No	No	Pathological (mild)	No
	18	24	1	3	0	1	5	Forced abdominal (moderate)	Yes	Yes	Physiological	No
	24	16	3	2	1	1	7	Forced abdominal (moderate)	No	Yes	Pathological (mild)	Yes
severe EA (n=7)	2	24	2	2	2	1	7	Forced abdominal (moderate)	No	Yes	Pathological (moderate)	No
	3	24	2	2	4	1	9	Forced abdominal (moderate)	No	Yes	Pathological (severe)	Yes

	5	24	3	3	4	1	11	Forced abdominal (severe)	Yes	Yes	Pathological (severe)	Yes
	6	12	3	1	1	1	6	Forced abdominal (severe)	No	Yes	Pathological (mild)	No
	7	20	3	2	1	1	7	Costo-abdominal	Yes	Yes	Pathological (mild)	Yes
	9	16	2	1	1	1	5	Forced abdominal (moderate)	Yes	Yes	Pathological (mild)	No
	10	16	2	1	1	1	5	Costo-abdominal	No	Yes	Pathological (moderate)	No

Additional File 3 (PDF): Results from the clinical examination and clinical scoring of the individual horses grouped in diagnoses. (Abbr.: EA=equine asthma, RR=respiratory rate)
