# Supplementary material for: Clinical variability of equine asthma phenotypes and analysis of diagnostic steps in phenotype differentiation
Source: Acta Vet Scand. 2024 Sep 18;66:51. doi: 10.1186/s13028-024-00773-7 (PMC11409572; doi:10.1186/s13028-024-00773-7)
Supplement: Supplementary file 4 — Additional file 4. Endoscopy and BALF cytology results. Horses and results are grouped according to their diagnoses. A total of 400 cells were counted per horse, expect from ID 14, in which the sample of BALF 1 was not sufficient and 300 cells were counted in BALF 2. The results from BALF 1 and 2 were pooled to obtain the results displayed in this table. (Abbr.: EA=equine asthma, BALF=bronchoalveolar lavage fluid, Macro=macrophages, Lymph=lymphocytes, MC=mast cells, Eos=eosinophils, GMC=giant multinucleated cells). [file 13028_2024_773_MOESM4_ESM.pdf]

Diagnosis	ID	Secretion Score	Viscosity Score	Swelling Score	Endoscopy Score (overall)	BALF recovered (mL)	Cells/µL BALF	Macro. (%)	Lymph. (%)	MC (%)	Neutrophils (%)	Eos. (%)	GMC (%)
Healthy (n=8)	*4*	0	0	0	0	290	32.5	58	37	0	3	0.5	1
	15	1	1	1	3	245	83.75	16.5	76	2.5	3.5	0.5	1
	19	1	1	0	2	210	54.25	56.5	27	3.75	7.25	0	5.5
	21	1	2	1	4	370	370	23.5	71.5	3	2	0	0
	22	2	2	0	4	330	71.25	49	41.5	2.5	5.5	0	1.5
	23	0	0	0	0	330	206.25	14.75	80.5	2.75	2	0	0
	25	2	1	1	4	410	128.75	26.25	67.75	1.5	3.75	0	0.75
	26	1	2	1	4	405	61.25	19.25	77.25	2.25	0.75	0.5	0
mild EA (n=4)	*11*	2	1	0	3	300	117.5	28.5	52.5	8.5	10	0.5	0
	13	2	1	2	5	275	60	49.75	29.25	5.75	15	0.25	0
	17	2	1	1	4	230	546.25	33	45.25	8.25	13.5	0	0
	20	1	1	1	3	200	68.75	50.5	42	6	1.5	0	0
moderate EA (n=7)	*1*	0	0	0	0	250	92.5	48	21	5	25.5	0.5	0
	8	2	2	0	4	405	62.5	46.5	38	5.75	7.5	2	0
	12	4	2	3	9	190	25	35.25	50	2	12.5	0	0.25
	14	1	2	0	3	180	61.25	58.3	18.6	2	21	0	0
	16	2	2	2	6	225	42.5	34	39.75	8	18.25	0	0
	18	2	3	2	7	230	87.5	35	41.75	8.25	10	4.5	0.5
	24	2	2	2	6	330	230	40.25	40.75	1.5	15	0	2.5
Severe EA (n=7)	*2*	3	2	0	5	205	238.75	23.5	21.5	0.5	54.5	0	0
	3	3	3	1	7	210	125	20	42	0	37.5	0	0.5
	5	4	3	2	9	175	227.5	35	15.5	0	49	0	0
	6	3	2	0	5	180	107.5	22.25	37.75	0.75	38.25	0	1
	7	3	2	2	7	245	122.5	45.5	18.5	1.5	34.25	0	0.25
	9	3	2	0	5	255	137.5	15	53.25	1.25	30.5	0	0
	10	2	3	2	7	350	143.75	33	18.75	1.5	46.75	0	0

Additional File 4 (PDF): Endoscopy and BALF cytology results. Horses and results are grouped according to their diagnoses. A total of 400 cells were counted per horse, expect from ID 14, in which the sample of BALF 1 was not sufficient and 300 cells were counted in BALF 2. The results from BALF 1 and 2 were pooled to obtain the results displayed in this table. (Abbr.: EA=equine asthma, BALF=bronchoalveolar lavage fluid, Macro=macrophages, Lymph=lymphocytes, MC=mast cells, Eos=eosinophils, GMC=giant multinucleated cells).
