# Supplementary material for: Clinical variability of equine asthma phenotypes and analysis of diagnostic steps in phenotype differentiation
Source: Acta Vet Scand. 2024 Sep 18;66:51. doi: 10.1186/s13028-024-00773-7 (PMC11409572; doi:10.1186/s13028-024-00773-7)
Supplement: Supplementary file 5 — Additional file 5. Arterial blood gas analysis. Results are sorted by ID as horses were presented to the clinic. The arterial partial pressure of O2 and CO2 were measured and the alveolar-arterial oxygen gradient was calculated in all horses. The mean group values for PaO2 were 101.79 mmHg (± 5.93) for healthy horses, 103.15 mmHg (± 2.55) for miEA, 97.21 mmHg (± 6.23) for modEA, and 82.75 mmHg (± 10.03) for sEA. The group means for PaCO2 were 42.49 mmHg (± 2.43) for healthy horses, 42.27 mmHg (± 1.84) for miEA, 43.44 mmHg (± 3.54) for modEA, and 43.80 mmHg (± 4.98) for sEA. The group means for AaD 02 were 1.2 mmHg (± 4.4) for healthy horses, -0.4 mmHg (± 2.8) for miEA, 4.3 mmHg (± 4.1) for modEA and 18.5 mmHg (± 7.6) for sEA. (Abbr.: EA=equine asthma, miEA=mild EA, modEA=moderate EA, sEA=severe EA, PaO2=arterial oxygen partial pressure, PaCO2=arterial carbon dioxide partial pressure, AaDO2=Alveolar-arterial oxygen gradient). [file 13028_2024_773_MOESM5_ESM.pdf]

Horse ID	PaO_2 (mmHg)	$PaCO_2$ (mmHg)	AaD 0_2 (mmHg)
1	96	46.1	2.9
2	79.1	40.9	25
3	84.3	39.9	20.8
4	112	38.6	-5.6
5	67	54.2	23.8
6	80.9	44.1	20
7	84.5	40.5	20
8	104	40.4	0.6
9	82.5	45.2	17.4
10	101	41.8	2.2
11	105	43.4	-3.4
12	102	38.5	4.5
13	103	39.7	2.3
14	94.1	44.6	6.3
15	105	39.3	0.7
16	104	42.8	-1.8
17	105	42.2	-2.2
18	92.2	42.6	10.2
19	99.4	43.1	2.5
20	99.6	43.8	1.6
21	105	45.1	-5.1
22	97.5	43.8	3.7
23	94.9	43.2	6.9
24	88.2	49.1	7.7
25	95.5	45.0	4.5
26	101	41.8	2.2

Additional File 5 (PDF): Arterial blood gas analysis. Results are sorted by ID as horses were presented to the clinic. The arterial partial pressure of O_2 and CO_2 were measured and the alveolar-arterial oxygen gradient was calculated in all horses. The mean group values for PaO_2 were 101.79 mmHg (± 5.93) for healthy horses, 103.15 mmHg (± 2.55) for miEA, 97.21 mmHg (± 6.23) for modEA, and 82.75 mmHg (± 10.03) for sEA. The group means for $PaCO_2$ were 42.49 mmHg (± 2.43) for healthy horses, 42.27 mmHg (± 1.84) for miEA, 43.44 mmHg (± 3.54) for modEA, and 43.80 mmHg (± 4.98) for sEA. The group means for AaD 0_2 were 1.2 mmHg (± 4.4) for healthy horses, -0.4 mmHg (± 2.8) for miEA, 4.3 mmHg (± 4.1) for modEA and 18.5 mmHg (± 7.6) for sEA. (Abbr.: EA=equine asthma, miEA=mild EA, modEA=moderate EA, sEA=severe EA, PaO_2=arterial oxygen partial pressure, $PaCO_2$=arterial carbon dioxide partial pressure, $AaDO_2$=Alveolar-arterial oxygen gradient)
