# Supplementary material for: Clinical variability of equine asthma phenotypes and analysis of diagnostic steps in phenotype differentiation
Source: Acta Vet Scand. 2024 Sep 18;66:51. doi: 10.1186/s13028-024-00773-7 (PMC11409572; doi:10.1186/s13028-024-00773-7)
Supplement: Supplementary file 6 — Additional file 6. Results from the blood examinations grouped in diagnoses. All values were in normal limits and group comparisons did not reveal any significant group differences. Dunnett’s post-hoc test of total plasma protein (measured via refractometry) showed a significant difference between healthy and sEA (adj. P=0.02), which may be attributed to the trend of higher fibrinogen levels in sEA horses (adj. P=0.0591). There was also a non-significant trend of lower eosinophils with disease severity (healthy vs. sEA, adj. P=0.0777). However, since results were all within the reference values and group sizes limited, findings do not seem to be beneficial for clinical diagnostics. (Abbr.: Adj. P = adjusted P-value, EA=equine asthma, sEA=severe EA). [file 13028_2024_773_MOESM6_ESM.pdf]

Additional File 6 (PDF): Results from the blood examinations grouped in diagnoses. All values were in normal limits and group comparisons did not reveal any significant group differences. Dunnett's post-hoc test of total plasma protein (measured via refractometry) showed a significant difference between healthy and sEA (adj. P=0.02), which may be attributed to the trend of higher fibrinogen levels in sEA horses (adj. P=0.0591). There was also a non-significant trend of lower eosinophils with disease severity (healthy vs. sEA, adj. P=0.0777). However, since results were all within the reference values and group sizes limited, findings do not seem to be beneficial for clinical diagnostics. (Abbr.: Adj. P = adjusted P-value, EA=equine asthma, sEA=severe EA)

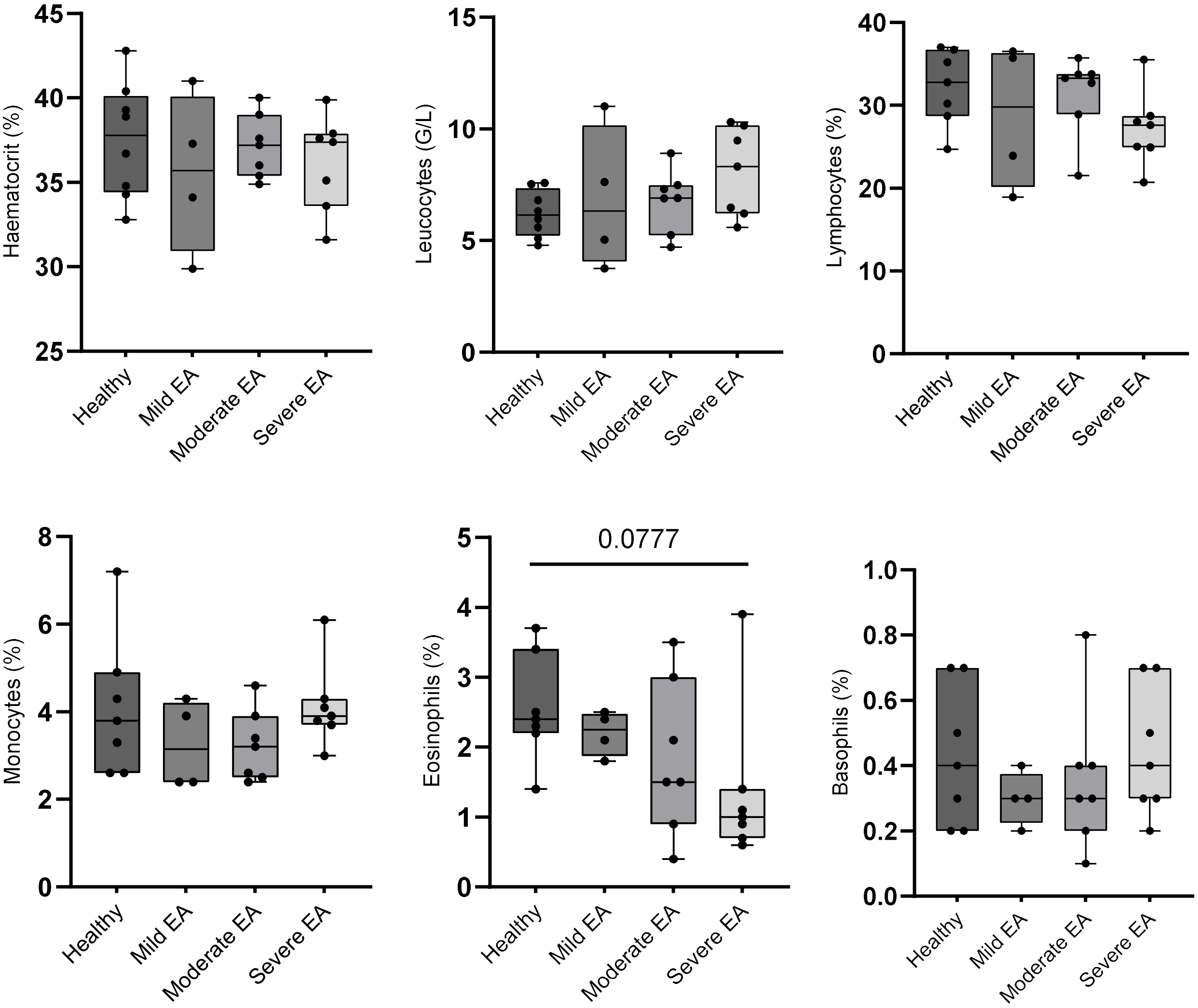

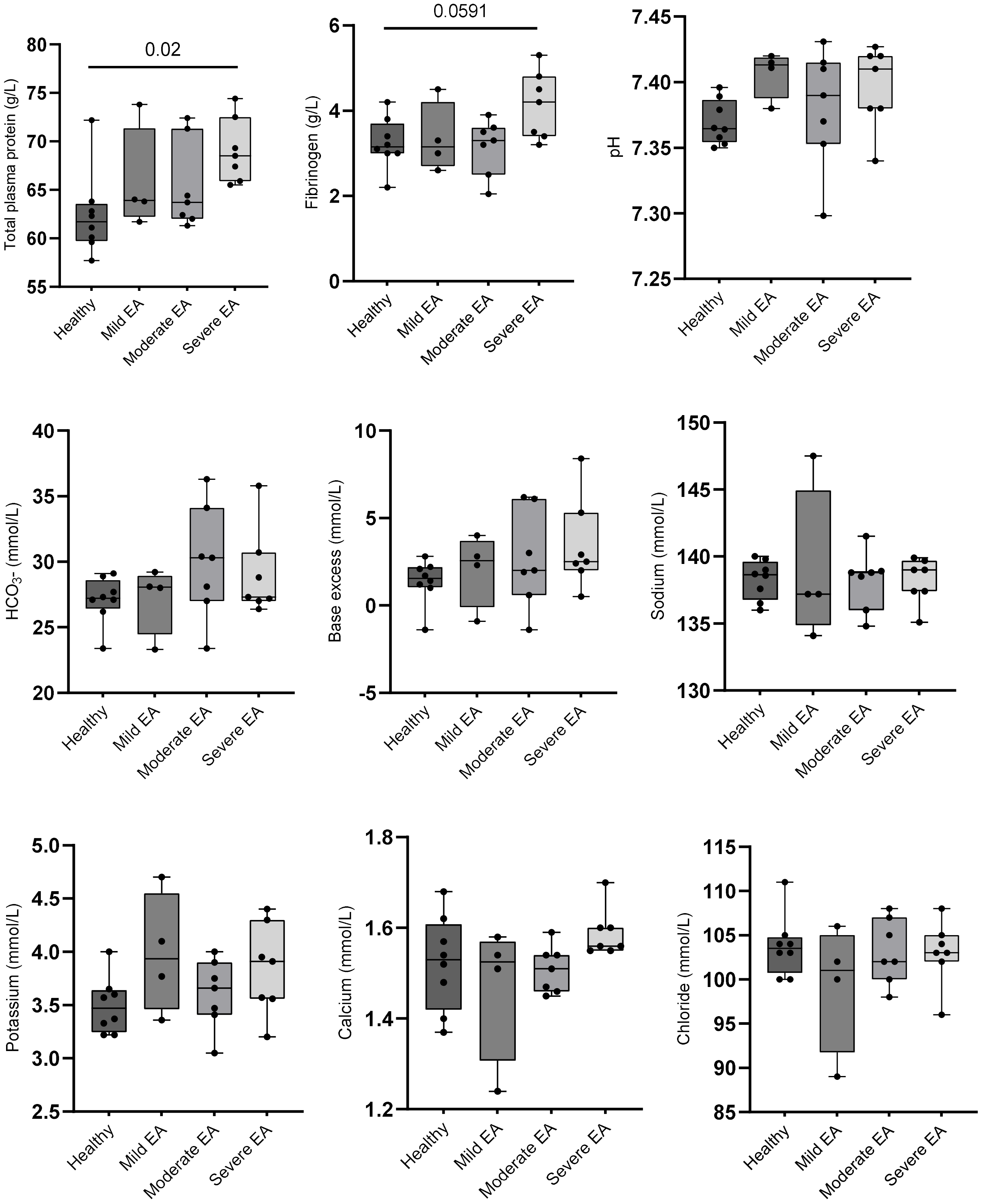

Adj. P=Dunnett's test for multiple comparisons
